# The Bacterial Type III Secretion System as a Broadly Applied Protein Delivery Tool in Biological Sciences

**DOI:** 10.3390/microorganisms13010075

**Published:** 2025-01-03

**Authors:** Liyu Jia, Lihua Zhu

**Affiliations:** 1 College of Forestry and Grassland, Nanjing Forestry University, Nanjing 210037, China; 15610321553@163.com; 2 Co-Innovation Center for Sustainable Forestry in Southern China, Nanjing Forestry University, Nanjing 210037, China

**Keywords:** type III secretion system (T3SS), protein secretion, crop applications, hypersensitive response, effector protein translocation

## Abstract

The type III secretion system (T3SS) is a nano-machine that allows Gram-negative bacteria to alter eukaryotic host biology by directly delivering effector proteins from the bacterial cytoplasm. Protein delivery based on the bacterial T3SS has been widely used in research in biology. This review explores recent advancements in the structure and function of the T3SS. We explore the molecular underpinnings of the T3SS apparatus, which spans bacterial and host cell membranes, and discuss the intricate transport mechanisms of effector proteins. Furthermore, this review emphasizes the innovative applications of the T3SS in crop biology, where it has been leveraged to study plant–pathogen interactions. By summarizing the current knowledge and recent progress, we underscore the potential of the T3SS as a powerful tool in biological sciences and their implications for future research in plant pathology and agricultural biotechnology.

## 1. Introduction

Bacterial protein secretion is a crucial aspect of the interaction between bacteria and their hosts [1]. However, bacterial protein secretion is limited by the plant’s defense responses. To overcome the plant cell membrane, bacteria have developed seven specialized secretion systems (types I to VII), with five spanning the bacterial double membrane [2]. Among these, the T3SS is a complex and well-studied system that directly delivers effector proteins from Gram-negative bacteria’s cytoplasm into eukaryotic organisms [3]. Since the discovery of bacterial T3SS over three decades ago, it has garnered widespread attention due to its role in effector delivery [4,5].

Pathogens use an array of effector proteins to facilitate the invasion of host plants [6,7,8,9]. Biotrophic plant pathogens establish disease by overcoming plant basal defenses [10], and the current theory suggests that this is due to the action of secreted effector proteins. Identification of the avirulence (*Avr*) genes of pathogenic bacteria will improve our understanding of the evolution of virulence and aid in the isolation of new resistance (*R*) genes. In recent years, the genomic sequences of various pathogens have been published, facilitating the identification of a wide array of secreted effector proteins. Common methods for studying effector proteins or avirulence genes include *Agrobacterium*-mediated gene expression [11], protoplast transformation [12,13], and particle bombardment [14]. However, the screening and functional analysis of potential effector proteins can be a time-consuming and labor-intensive process. The T3SS of a bacterial plant pathogen can be used as an alternative method for effector or *Avr* screening in plant cells. This innovative approach streamlines the process and offers a valuable tool for advancing our understanding of plant–pathogen interactions and for the development of disease resistance in crops.

The T3SS, therefore, is not only a valuable tool for studying plant–pathogen interactions but also holds significant promise for the development of novel strategies in plant disease management and resistance breeding. Research reports indicate that the N-terminal signal sequences of AvrRpm1, AvrRps4, and AvrBs2, when fused with effector proteins, can be successfully recognized and delivered into plants such as wheat, rice, Arabidopsis, tobacco, potato, pepper, and tomato through the T3SS [8,15,16,17,18,19,20,21].The ability to deliver specific genes into plants using the T3SS could lead to innovative approaches for enhancing crop resilience against diseases. As research continues to optimize the T3SS for improved gene delivery and expression in plants, it enhances our ability to manipulate plant–pathogen interactions and resistance breeding in agriculture. This article provides an overview of the T3SS’s architecture, its mechanisms of effector protein translocation and secretion, and its applications in crop systems.

## 2. Structure of Bacterial Type III Secretion System

The T3SS is a molecular syringe used by Gram-negative pathogens to inject effector proteins into host cells and represents an ideal conduit due to its non-essential nature for pathogen growth [22,23,24]. This advanced system is used by various human pathogens, such as *Chlamydia*, *EPEC*, *Pseudomonas aeruginosa*, *Salmonella*, *Shigella*, and *Yersinia*, which use the T3SS to subvert host cell functions and establish infection [25]. Similarly, plant pathogens such as *Erwinia*, *Pseudomonas syringae*, and *Xanthomonas*, as well as plant symbionts like *Rhizobium*, rely on T3SS injectisomes to manipulate plant cells, to promote disease, or to establish beneficial symbiotic relationships [26,27,28,29]. The T3SS apparatus, a needle-like structure, spans various bacterial membranes, allowing for the direct translocation of bacterial effector proteins into host cells and establishing these effectors within the host cell membrane, facilitating the establishment of bacterial resistance [24,30].

**Figure 1:**
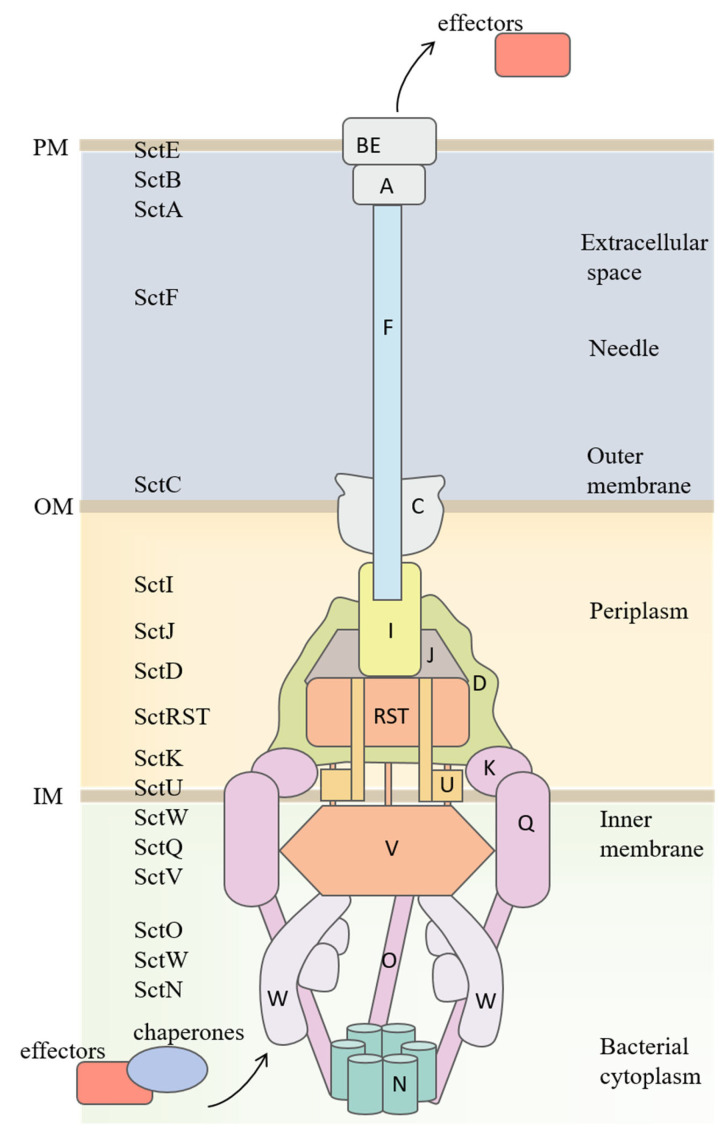
Structure of the T3SS in bacteria. The T3SS components are identified using the unified nomenclature Sct names {secretion and cellular translocation [44]}. The cartoon represents the secretion state of the effector protein. OM, outer membrane; PM, plant membrane; IM, inner membrane.

The T3SS is a complex molecular syringe comprising membrane structure apparatus and translocator proteins that span bacterial and host cell membranes [31]. This system is divided into seven parts, extending from the bacterium to the host cell, which includes the ATPase complex, C-ring, secretion apparatus, basal body, needle complex, tip complex, and translocon pore [22,32,33]. The basal body is a set of ring-like structures within bacterial membranes that serve as a stable platform for the secretion apparatus [33,34,35,36]. The secretion apparatus, situated at the base of the basal body, comprises five membrane proteins: SctR, SctS, SctT, SctU, and SctV [37]. The secretion apparatus is located below the C-ring, consisting of SctQ and the ATPase complex, which includes ATPase-SctN, SctO, SctL, and SctK. These components serve as a platform for substrate recruitment and secretion [38,39]. In the T3SS of some bacteria, a needle extends from the surface of the bacterium and is externally enveloped by the SctA tip complex [40,41]. The tip complex facilitates the assembly of the translocator proteins SctE and SctB into a translocon pore in the host cell membrane, establishing a channel between the bacterial cytoplasm and the host cell cytoplasm, which allows effectors to be directly translocated (Figure 1).

The entire T3SS apparatus is also referred to as an “injection apparatus”. The T3SS needle allows bacteria to inject effectors into host cells, creating an environment conducive to colonization [42,43]. The advanced nanomachine is a significant target for therapeutic intervention due to its significant role in the pathogenesis of various diseases.

## 3. Mechanism of Effector Protein Transport

The secretins of the T3SS are composed of three N-terminal N-domains (N0, N1, and N3) that extend into the periplasm, a C-domain that serves as the outer membrane entry point, and an S-domain where a specific piloting protein binds to the secretin, facilitating its localization and formation [45]. The secreted effectors, although diverse in function and structure, are all secreted through a common ATP and PMF-driven one-step mechanism, involving an N-terminal signal and often targeting chaperones [29].

The T3SS plays a crucial role in facilitating the translocation of effector proteins from bacteria to host cells. The process is initiated by the recognition of the N-terminal secretion signal (NSS) on the effector protein or through the assistance of a chaperone binding domain (CBD) [46,47,48]. The NSS is a short sequence located at the N-terminus of type III secretion system effectors (T3Es). One of its primary functions is to be recognized by the T3SS, thereby facilitating the translocation of effector proteins to their target destination. The secretion and translocation of effector proteins are tightly regulated processes. Chaperone proteins are essential for maintaining unfolded effector proteins, preparing them for secretion, and in some cases, for the recognition and binding of the effector to the T3SS. Effector proteins once translocated into the host cell, can manipulate cellular processes to facilitate pathogen survival and colonization.

Researchers have discovered that effector proteins can be secreted with just an NSS, such as the YopE and YopH effectors in Yersinia, which require only the first 15 and 17 amino acid residues, respectively, for secretion into the bacterial extracellular matrix [49]. The NSS differs from the signal peptide (Sec), as the NSS of T3SS effectors does not have a consistent N-terminal sequence [50], and there is no conserved tertiary structure [51].

**Figure 2:**
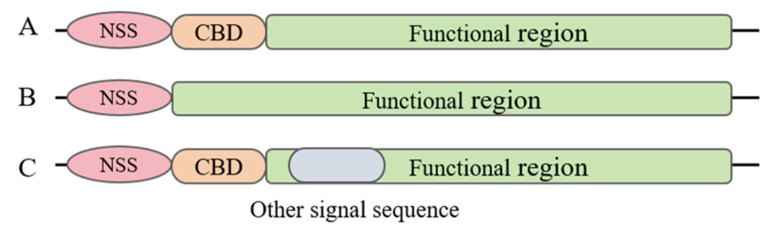
Types of effector proteins secreted by the T3SS. (**A**) Effector containing NSS and CBD; (**B**) effector containing NSS alone; (**C**) effector containing NSS, CBD, and other signal sequences.

Some effector proteins require not only the NSS for translocation but also the assistance of a CBD. The CBD, located amino-terminal to the NSS, is the region where the effector protein interacts with its chaperone [52,53]. Chaperones for the T3SS exist in the cytoplasm primarily as dimers, are typically small in size, and serve to maintain the stability of the effector proteins, assist in their translocation, and facilitate subsequent secretion [54]. The translocation of effector proteins is influenced not only by the NSS and CBD but also by other genetic elements of the effector proteins themselves. Chaperones play a crucial role in the T3SS, ensuring the proper folding, secretion, and translocation of effector proteins. Chaperones are proteins that bind to effector proteins, preventing their premature aggregation or degradation within the bacterial cell. Secretion signals and chaperone binding domains are crucial for the recognition and transport of effector proteins by the T3SS apparatus, a key virulence factor in numerous Gram-negative pathogens. In summary, the T3SS secretes effector proteins, which can be categorized into three types (Figure 2).

The T3SS controls the secretion and transfer of effector proteins by recognizing the NSS with the secretion machinery. This process is crucial for the pathogen to manipulate host cell functions and initiate infection. The discovery that a minimal NSS is sufficient for effector secretion highlights the versatility and adaptability of T3SS in various bacterial pathogens.

## 4. Application of Bacterial Type III Secretion Systems to Crops

The T3SS recognizes genes with a type III effector N-segment recognition signal and delivers the gene as a protein to the cytoplasm of the plant. A diverse array of effector proteins has been effectively delivered into various crop species, leveraging this theoretical approach.

### 4.1. Application of Bacterial Type III Secretion Systems to Arabidopsis Cells

**Figure 3:**
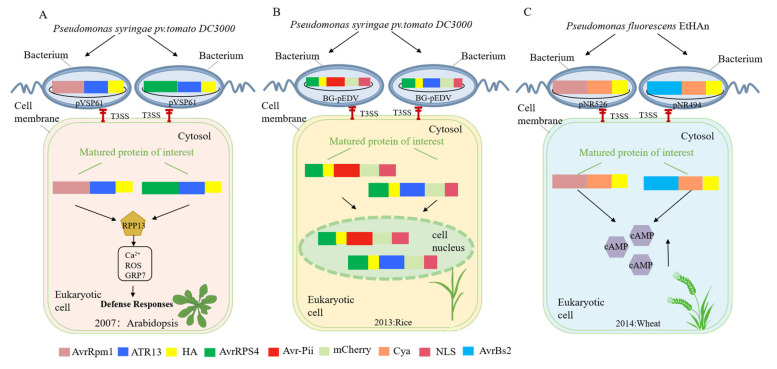
Application of bacterial type III secretion systems to crops. (**A**) The T3SS recognizes ATR13 fused to the NSS of AvrRpm1 or AvrRps4 and translocates it into Arabidopsis thaliana cells; (**B**) the T3SS recognizes *AvrPii* and *AvrPik* fused to the NSS of AvrRps4 and translocates it into rice cells; (**C**) the T3SS recognizes Cya fused to the NSS of AvrRpm1 or AvrBs2 and translocates it into wheat cells.

T3SS successfully recognizes oomycete effectors with N-terminal signal sequences of bacterial effectors and delivers them to Arabidopsis cells [17,19,55] (Figure 3A). In 2007, Rentel and his team have developed a genetically effective system for detecting the recognition of ATR13-RPP13 [19]. This system used the bacterium *P. syringae* pv. *tomato* DC3000 (*Pst*) and involved fusing ATR13 with the N-terminal signal sequence (NSS) of AvrRpm1. By deploying ATR13 via *Pst*, the NSS of AvrRpm1 was able to translocate ATR13 through the T3SS into the host cells. This innovative approach facilitated the study of effector protein delivery and host–pathogen interactions in plants [56,57].

The same year, Sohn et al. discovered that *Pst* DC3000 efficiently delivers bacterial (AvrRps4) and oomycete (ATR1 and ATR13) effector chimeras to plant cells in the same year. These chimeric effector proteins triggered effector-triggered immunity (ETI) in Arabidopsis plants carrying the corresponding *RPP* genes (*RPP1*-*Nd*/*WsB* or *RPP13-Nd*) [17]. The study demonstrated that the delivery of ATR1 and ATR13 via T3SS could elicit a robust immune response, providing valuable insights into the molecular mechanisms underlying plant–pathogen interactions. The study demonstrated that the presence of multiple ATR1 and ATR13 alleles can enhance the virulence of *Pst* DC3000 in susceptible Arabidopsis accessions, highlighting their role in promoting pathogen virulence within host cells.

### 4.2. Application of Bacterial Type III Secretion Systems to Rice Cells

The T3SS has been successfully employed to identify and deliver fungal effector proteins with bacterial N-terminal signal sequences into rice cells [8] (Figure 3B). Prior research has indicated that oomycete effector proteins can be transmitted via the T3SS of *Pst* in dicotyledonous plants using pEDV [19,58]. *Burkholderia glumae* (*Bg*), a Gram-negative bacterium, is responsible for various diseases in rice, including rice panicle blight, grain rot, sheath rot, and seedling rot [59]. In 2013, Sharma and colleagues revealed that the release of *Avr-Pik* and *Avr-Pii* through the *Bg* T3SS into rice varieties containing the corresponding R genes resulted in the inhibition of *Bg* growth. Concurrently, the delivery of ATR1 and ATR13 avirulence or non-toxic genes into susceptible or resistant Arabidopsis through the T3SS allowed for the assessment of Arabidopsis resistance to ATR1 and ATR13 by monitoring the proliferation of *P. syringae*.

### 4.3. Application of Bacterial Type III Secretion Systems to Wheat Cells

The T3SS has successfully identified fungal effectors harboring bacterial effector N-terminal signal sequences and can effectively deliver them into wheat cells [21] (Figure 3C). In their pioneering study, Upadhyaya and colleagues engineered a fusion of the NSS of AvrRpm1 and AvrBs2 to the protein calmodulin-dependent adenylate cyclase (Cya), leveraging the bacterial T3SS to translocate the chimeric protein into wheat leaf cells. The efficacy of this delivery system was confirmed by monitoring the cAMP enzyme activity within the wheat leaf cells, which served as a biomarker for the successful introduction of the gene construct.

The T3SS has been successfully applied to deliver pathogenic effector proteins into various crop species beyond Arabidopsis, rice, and wheat. In 2014, Upadhyaya and colleagues demonstrated the utility of T3SS in delivering AvrBs2 and AvrPto, effector proteins from *Xanthomonas campestris* pv. *vesicatoria* (*Xcv*) and *Pseudomonas syringae* pv. *tomato* (*Pst*), respectively, into pepper and tomato plants [15,18,21]. Furthermore, the T3SS has been instrumental in the delivery of the *DspA/E* effector proteins from the fire blight pathogen, *Erwinia amylovora*, into tobacco cells [16,20].

The studies significantly enhanced our comprehension of the T3SS, its role in delivering bacterial effector proteins into plant cells, and its implications for disease resistance breeding.

## 5. Conclusions and Future Directions

In this review, we focus on the structural characteristics of the bacterial T3SS and the prerequisites for its application in delivering various effector proteins, and we present an overview of current case studies where T3SS has been applied in crops.

The T3SS is a sophisticated mechanism utilized by certain bacteria to deliver effector proteins directly into the cytoplasm of eukaryotic host cells [22,60]. This system has been harnessed for various applications in plant pathology and breeding for disease resistance. The T3SS is a nanosyringe-like structure that allows for the translocation of proteins that can manipulate host cell processes to the pathogen’s advantage [22,29].

The T3SS is used in agriculture to deliver effector proteins to various crop species. Studies have successfully used the T3SS to translocate proteins into monocotyledonous plants such as wheat, rice, and barley, which were previously thought to be less susceptible to such methods due to their unique cell wall structure [61]. This has been achieved by fusing the N-terminal signal sequence of bacterial type III effectors, such as AvrRpm1, AvrRps4, and AvrBs2, to the proteins of interest. The T3SS detects signal sequences and delivers fused proteins into plant cells, eliciting responses or studying plant–pathogen interactions [8,15,16,17,18,19,20,21].

The T3SS is a multifaceted molecular structure present in various Gram-negative bacterial pathogens, including *Pseudomonas*, *Yersinia*, *Shigella*, *Salmonella*, and pathogenic *E. coli*, among others [62]. *Pseudomonas* species, in particular, are well-studied plant pathogens that provide insights into host–microbe interactions, bacterial virulence mechanisms, host adaptability, and the evolution and epidemiology of microbial pathogens [63]. The *Pseudomonas fluorescens* strain *Et*HAn has been shown to possess a functional T3SS that can secrete effectors but not translocate them into host cells, making it a potential tool for studying plant–pathogen interactions without causing necrosis [21]. Other strains, such as *P. syringae* pv. *maculicola* strain *Psm*ES4326 and *Pst* DC3000, have been extensively studied for their T3SS and its role in pathogenicity [17,19,23,42,64]. The T3SS in these bacteria includes a complete set of functional genes for the secretion system and does not cause plant necrosis, which makes it possible to use these systems for high-throughput screening of candidate effectors.

The T3SS successfully translocates effector proteins into eukaryotic cells, relying on the NSS of bacterial type III effectors, according to research [51]. It has been observed that different NSSs are required for the recognition and delivery of these effectors in various plant species. For instance, the NSS of AvrRps4 is not effective in wheat but works in rice and Arabidopsis [8,19,21]. The efficiency of different bacterial NSSs in delivering fused genes also varies, highlighting the importance of selecting or modifying the NSS for optimal gene expression in crops. The study suggests that future research could improve the efficiency of gene delivery and expression in plants using the T3SS.

However, the T3SS has limitations in the size and structure of the proteins it can transport. For instance, the T3SS struggles to deliver complexly folded domains, such as the tightly packed green fluorescent protein (GFP) domain, making the secretion and translocation of large fluorescent proteins like GFP quite challenging [61]. The T3SS faces constraints when transporting larger genes, although the upper limit of the gene size that can be transported is not yet clearly understood.

The T3SS is a versatile tool that aids in studying plant–pathogen interactions and devising strategies to improve crop disease resistance [22]. The T3SS facilitates the delivery of bacterial effector proteins into plant cells, allowing researchers to study pathogen virulence and plant defense mechanisms, potentially identifying new breeding targets. This system has been effectively applied across a range of plant species, demonstrating its potential to probe plant–pathogen interactions and to develop targeted therapeutics in agriculture.

## Data Availability

Data are contained within the article.
